# Evidence for the contribution of *HCN1* gene polymorphism (rs1501357) to working memory at both behavioral and neural levels in schizophrenia patients and healthy controls

**DOI:** 10.1038/s41537-022-00271-7

**Published:** 2022-08-20

**Authors:** Xiongying Chen, Qiumei Zhang, Yanyan Su, Wan Zhao, Yang Li, Boqi Du, Xiaoxiang Deng, Feng Ji, Qi Dong, Chuansheng Chen, Jun Li

**Affiliations:** 1 The National Clinical Research Center for Mental Disorders & Beijing Key Laboratory of Mental Disorders, Beijing Anding Hospital & the Advanced Innovation Center for Human Brain Protection, Capital Medical University, Beijing, P.R. China; 2 School of Public Health, Jining Medical University, Jining, Shandong Province P.R. China; 3 State Key Laboratory of Cognitive Neuroscience and Learning & IDG/McGovern Institute for Brain Research, Beijing Normal University, Beijing, P.R. China; 4 School of Psychology, Nanjing Normal University, Nanjing, P.R. China; 5 Department of Psychological Science, University of California, Irvine, CA USA

**Keywords:** Working memory, Schizophrenia

## Abstract

Gene HCN1 polymorphism (rs1501357) has been proposed to be one of the candidate risk genes for schizophrenia in the second report of the Psychiatric Genomics Consortium–Schizophrenia Workgroup. Although animal studies repeatedly showed a role of this gene in working memory, its contribution to working memory in human samples, especially in schizophrenia patients, is still unknown. To explore the association between rs1501357 and working memory at both behavioral (Study 1) and neural (Study 2) levels, the current study involved two independent samples. Study 1 included 876 schizophrenia patients and 842 healthy controls, all of whom were assessed on a 2-back task, a dot pattern expectancy task (DPX), and a digit span task. Study 2 included 56 schizophrenia patients and 155 healthy controls, all of whom performed a 2-back task during functional magnetic resonance imaging (fMRI) scanning. In both studies, we consistently found significant genotype-by-diagnosis interaction effects. For Study 1, the interaction effects were significant for the three tasks. Patients carrying the risk allele performed worse than noncarriers, while healthy controls showed the opposite pattern. For Study 2, the interaction effects were observed at the parietal cortex and the medial frontal cortex. Patients carrying the risk allele showed increased activation at right parietal cortex and increased deactivation at the medial frontal cortex, while healthy controls showed the opposite pattern. These results suggest that the contributions of rs1501357 to working memory capability vary in different populations (i.e., schizophrenia patients vs. healthy controls), which expands our understanding of the functional impact of the HCN1 gene. Future studies should examine its associations with other cognitive functions.

## Introduction

Schizophrenia (SCZ) is characterized by severe psychotic and cognitive symptoms. It afflicts ~1% population worldwide (GBD, 2016). Because of its high heritability (~80%), identifying its risk genes and figuring out their functions become an effective way to uncover the pathogenesis of SCZ. Previously, a large number of SCZ risk genes have been reported by genome-wide association studies (GWAS), with *HCN1* being one of the most promising genes. *HCN1* was firstly identified by the Psychiatric Genomics Consortium–Schizophrenia Workgroup (PGC–SCZ) in their second report (PGC2). In PGC2, an intron SNP (rs1501357) in *HCN1* gene was listed as the 76^th^ top risk polymorphism for SCZ^1^. Afterwards, a genome-wide association study in Han Chinese sample replicated the association between the same SNP and SCZ^2^. When Lencz and Malhotra^3^ further examined all the PGC2-reported SCZ risk genes together with the databases of all approved and in-trial pharmaceutical compounds for SCZ, *HCN1* was identified as one of the five potential target genes for the treatment of psychotic symptoms in SCZ (the others were *CACNA1C*, *CACNB2*, *CACNA1I*, *GRIN2A*). *HCN1* encodes a voltage-gated potassium/sodium channel that has been evidenced to be a main contributor to hyperpolarization-activated cation current. In addition to its role in regulating neuronal excitability, *HCN1* has also been found in animal studies to play a significant role in rhythmic activity, synaptic plasticity, etc.^4^.

However, the exact cognitive functions of *HCN1* are still largely unknown. Some previous animal studies have revealed that brain *HCN1* level may be associated with working memory (WM) in a non-linear pattern. Nolan et al.^5^ found that spatial memory of mice with *HCN1* deletion from its forebrain neurons was enhanced. Similarly, Wang et al.^6^ found that knockdown of *HCN1* channel in prefrontal cortex of rats improved WM performance at both behavioral and neural levels. By contrast, Thuault et al.^7^ reported that WM performance of rats was decreased but not improved when *HCN1* was only deleted from neurons spatially located in medial prefrontal cortex. Indeed, the functional significance of rs1501557 (e.g., its functional consequence on animal HCN channel expression/function or on human cognitive function) is still unknown.

Thus far, no human study has directly examined the relationship between *HCN*1 and WM. Indirect evidence has come from two recent studies that linked some common variants near *HCN1* to cognitive functions related to WM, such as spatial memory and educational attainment^8,9^. Specifically, using a total of 726 patients with SCZ and 1063 healthy controls, the Consortium on the Genetics of Schizophrenia (COGS) performed a GWAS to evaluate genetic associations with 11 neural and cognitive endophenotypes. The authors identified 9 regions showing significant or near significant associations, one of which included a SNP (rs187763903, ~290 kb upstream of *HCN1*) showing a significant association with spatial memory^8^. In the other study, Okbay et al.^9^ conducted a GWAS that involved a discovery sample of 293,723 healthy individuals and a replication sample of 111,349 healthy individuals and reported a SNP (rs4493682, ~100 kb upstream of *HCN1*) as one of the most significant SNPs that showed associations with educational attainment. The current study aimed to examine the association between the PGC2-identified SNP within *HCN1* (rs1501357) and WM in two independent human samples (a large sample for a behavioral study, and a smaller sample for an fMRI study). Both studies included patients with SCZ and healthy controls. We postulated that the SCZ risk allele ‘C’ of rs1501357 would be associated with worse WM performance at behavioral level and less efficient brain function during a WM task.

## Results

### Study 1: Effects of rs1501357 on working memory differed by diagnosis

Genotypes of rs1501357 did not show evidence of deviation from Hardy-Weinberg equilibrium (*P* > 0.05). No significant difference in demographic variables was observed across genotypes (TT vs. TC vs. CC, *P*s > 0.05, see Table 1).

**Table 1:** Demographic and clinical factors across rs1501357 genotypes for study 1.

	Mean ± SD			F or χ^2^	*P*
	TT	TC	CC		
Age					
Controls	26.73 ± 8.22	26.40 ± 8.18	25.53 ± 8.26	1.297	0.274
Patients	28.77 ± 7.73	28.40 ± 7.89	29.60 ± 7.79	1.705	0.182
Gender (male/female)					
Controls	100/83	208/226	119/106	3.138	0.208
Patients	129/78	284/169	138/78	0.129	0.938
Education (years)					
Controls	9.28 ± 3.37	9.43 ± 3.51	9.80 ± 3.13	1.408	0.245
Patients	9.94 ± 3.18	9.76 ± 3.10	9.12 ± 3.04	2.893	0.056
PANSS positive					
Patients	18.92 ± 6.87	18.33 ± 7.04	18.57 ± 7.11	0.296	0.744
PANSS negative					
Patients	17.27 ± 7.88	17.39 ± 7.94	17.81 ± 8.21	0.170	0.844
Medication dose (mg/day)^a^					
Patients	599.28 ± 381.64	651.90 ± 521.45	623.40 ± 437.92	0.743	0.476

^a^Chlorpromazine equivalents.

**Fig. 1:**
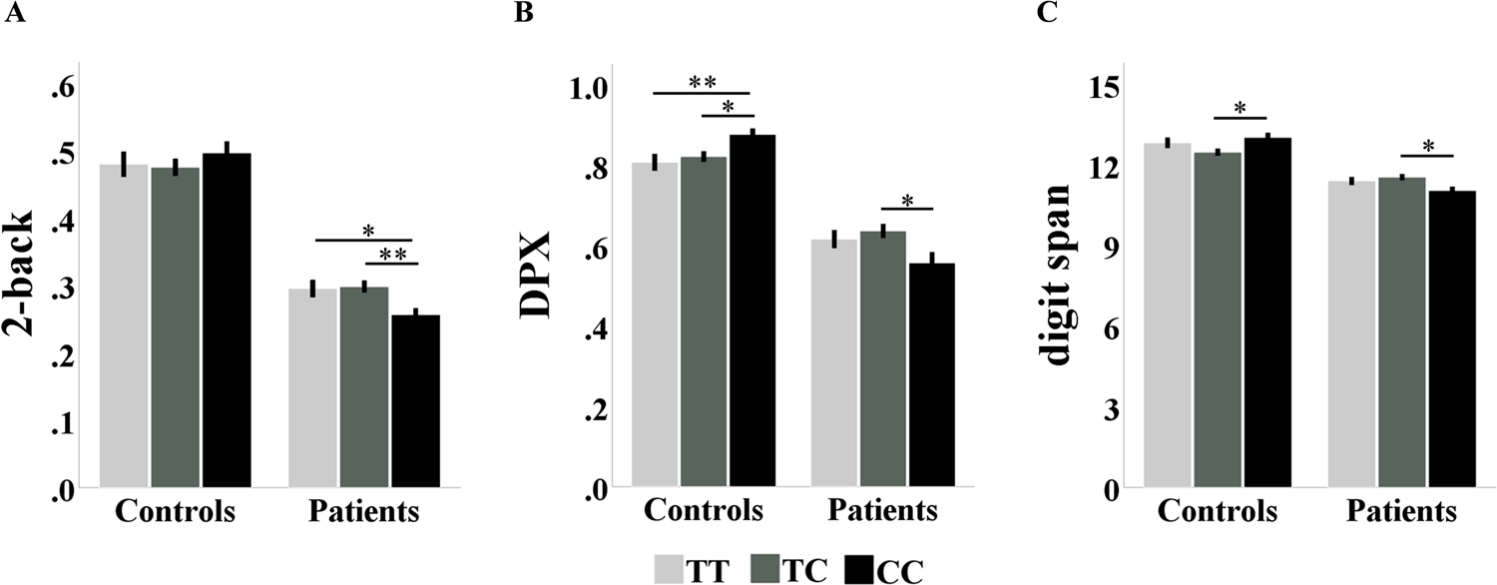
Genotype effects of rs1501357 on task performance in Study 1. **A**–**C** The 2-back, DPX and digit span task performance by genotype and diagnosis, respectively. *P < 0.05, **P < 0.01.

As shown in Table 2, ANOVA revealed significant genotype-by-diagnosis interaction effects on the performance of the DPX task (F = 7.208, *P* < 0.001), the digit span task (F = 6.776, *P* = 0.001), and the 2-back task (F = 3.057, *P* = 0.047). However, the main effect of genotype was not significant (DPX: F = 0.497, *P* = 0.609; digit span: F = 0.247, *P* = 0.781; 2-back: F = 0.360, *P* = 0.697). Post-hoc analyses found that the significant interaction effects were due to the opposite genotypic effects in patients and controls. In the DPX task, the risk ‘C’ allele showed a dose-dependent association with worse performance in patients (F = 2.582, *P* = 0.036, see Fig. 1B) but better performance in healthy controls (F = 4.325, *P* = 0.014). In the digit span task, the risk ‘C’ allele again showed association with worse performance in patients (F = 3.503, *P* = 0.031, see Fig. 1C) but better performance in the controls (F = 3.511, *P* = 0.030). For the 2-back task, the pattern was similar, although only the genotype effect in the patients reached significance (for patients, F = 4.244, *P* = 0.015; for controls, F = 0.516, *P* = 0.597, see Fig. 1A).

**Table 2:** Working Memory Performance Across rs1501357 Genotypes for Study 1.

	Mean ± SD (*n*)			F_diagnosis_(*P*)	F_genotypes_(*P*)	F_diagnosis×genotypes_(*P*)	F_simple effects_(*P*)
	TT	TC	CC				
2-back							
Controls	0.48 ± 0.25 (178)	0.48 ± 0.26 (415)	0.50 ± 0.26 (225)	304.58 (<0.001)	0.360 (0.697)	3.057 (0.047)	0.516 (0.597)
Patients	0.30 ± 0.18 (195)	0.30 ± 0.18 (418)	0.26 ± 0.14 (198)				4.244 (0.015)
DPX							
Controls	0.81 ± 0.29 (185)	0.82 ± 0.28 (427)	0.88 ± 0.22 (230)	204.50 (<0.001)	0.497 (0.609)	7.208 (<0.001)	4.325 (0.014)
Patients	0.62 ± 0.26 (138)	0.64 ± 0.31 (307)	0.56 ± 0.32 (138)				2.582 (0.036)
Digit span							
Controls	12.82 ± 2.57 (180)	12.47 ± 2.72 (430)	13.02 ± 2.56 (228)	127.54 (<0.001)	0.247 (0.781)	6.776 (0.001)	3.511 (0.030)
Patients	11.40 ± 2.09 (200)	11.54 ± 2.31 (433)	11.04 ± 2.14 (201)				3.503 (0.031)

### Study 2: SNP rs1501357 showed significant associations with working memory related brain activation

Genotypes of rs1501357 did not show evidence of deviation from Hardy-Weinberg equilibrium (*P* > 0.05). No significant difference between genotype groups (TT/TC vs. CC) was found for any demographic factors or task performance (*P*s > 0.05, see Table 3).

**Table 3:** Demographic and clinical factors across rs1501357 Genotypes for Study 2.

	Mean ± SD		F or χ^2^	*P*
	TT/TC	CC		
2-back				
Controls	0.90 ± 0.15	0.86 ± 0.20	1.789	0.183
Patients	0.73 ± 0.22	0.83 ± 0.11	2.821	0.099
Age				
Controls	26.89 ± 5.35	27.95 ± 5.92	1.110	0.294
Patients	28.28 ± 7.62	26.75 ± 8.36	0.433	0.513
Gender (male/female)				
Controls	87/27	30/11	0.060	0.807
Patients	35/5	15/1	4.742	0.316
Education (years)				
Controls	13.75 ± 3.17	12.71 ± 3.22	3.239	0.074
Patients	12.73 ± 3.22	11.81 ± 2.76	0.991	0.324
PANSS positive				
Patients	19.60 ± 6.68	21.50 ± 7.96	0.518	0.477
PANSS negative				
Patients	20.88 ± 6.57	17.20 ± 7.57	2.056	0.161
Medication Dose (mg/day)^a^				
Patients	633.13 ± 432.16	640.23 ± 516.47	0.392	0.535

^a^Chlorpromazine equivalents.

**Fig. 2:**
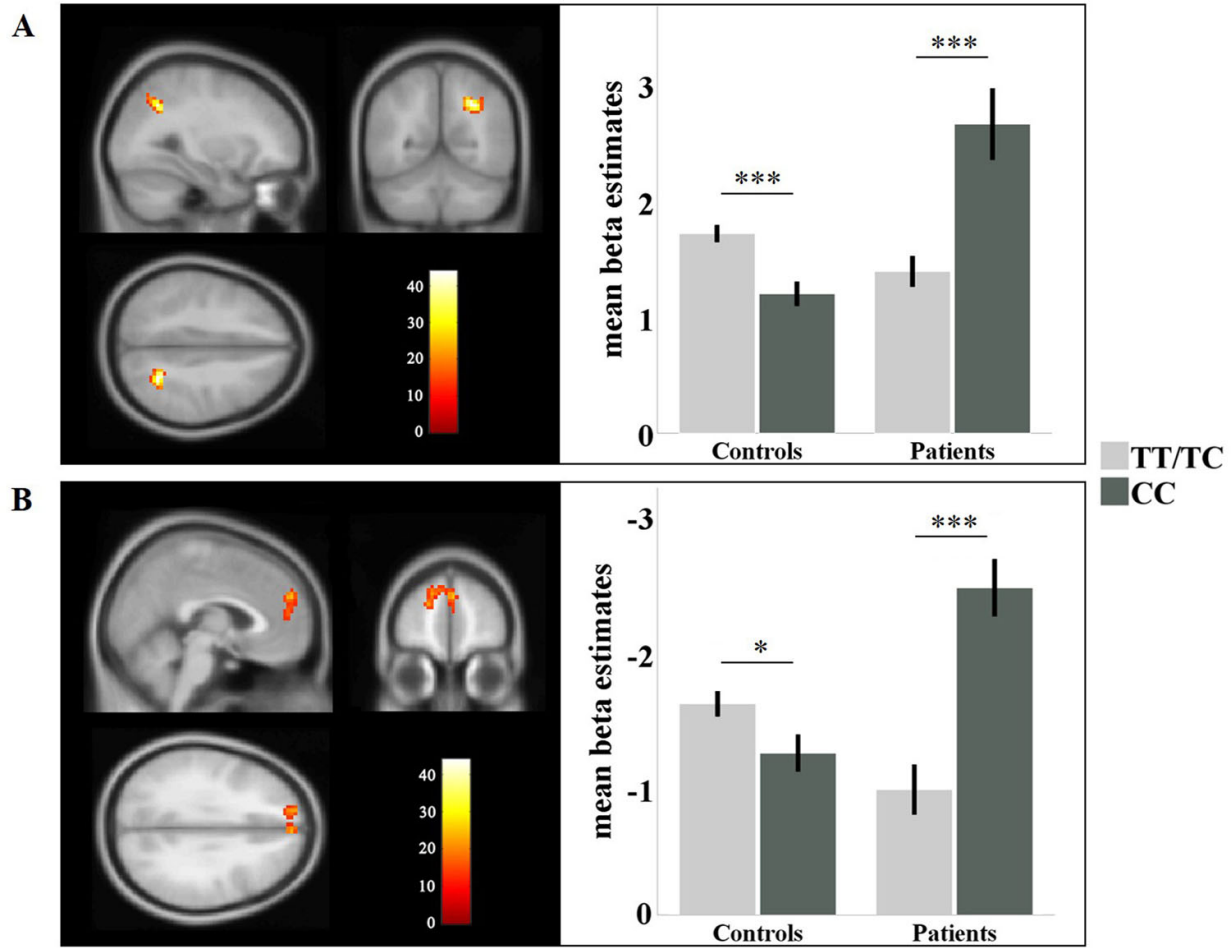
Genotype effects of rs1501357 on brain activation in Study 2. **A**, **B** The significant genotype-by-diagnosis interaction effects within the right parietal cortex and the medial prefrontal cortex in the n-back task, respectively. *P < 0.05, ***P < 0.001.

The full factorial ANOVA across the whole brain identified a significant genotype-by-diagnosis interaction effect within the right parietal cortex (cluster size = 90, F_max(90)_ = 40.39, *P* = 0.001, see Fig. 2A) and the medial frontal cortex (cluster size = 178, F_max(178)_ = 25.12, *P* < 0.001, see Fig. 2B). However, we did not find any significant main effect of genotype. This pattern was the same as that for behavioral data in Study 1. Post-hoc analyses were conducted to test the significant interaction effects in the two brain regions. In the right parietal cortex, a region that was activated during the n-back task, carriers of the risk “C” allele showed higher activation than non-carriers in patients did (F = 19.661; *P* < 0.001, see Fig. 2A), but lower activation than non-carriers in controls did (F = 14.139; *P* < 0.001, see Fig. 2A). In the medial frontal cortex, a region that was deactivated during the n-back task, “C” allele carriers showed greater deactivation in patients (F = 21.481; *P* < 0.001, see Fig. 2B) but less deactivation in controls (F = 4.283; *P* = 0.040, see Fig. 2B).

Because HCN channels govern the strength of prefrontal cortical connections in primates^6^, it would be important to see if the role identified in brain regions outside the prefrontal cortex (e.g., the parietal cortex) was due to functional connectivity with the prefrontal cortex. We then used the identified parietal cortex as a seed to conduct PPI analysis (see supplementary materials). However, this analysis did not show any significant result within the prefrontal cortex, which indicated that the functional impact of rs1501357 on the parietal cortex was independent of the functional connectivity with the prefrontal cortex.

## Discussion

Using two independent samples, this study for the first time examined the associations between *HCN1* gene polymorphism and WM at both behavioral and neural levels. At both levels, rs1501357 of *HCN1* showed an opposite pattern of association with WM in patients with SCZ and healthy controls. The risk “C” allele was associated with worse WM performance on all three tasks (the DPX, digit span, and n-back tasks) in patients but better WM performance on two tasks (the DPX and digit span tasks) in controls. At the neural level (based on the n-back task), “C” allele was associated with higher brain activation at the parietal cortex and greater deactivation at the medial prefrontal cortex in patients but lower brain activation at the parietal cortex and less deactivation at the medial prefrontal cortex in controls. These results were consistent with previous animal studies and also suggested an important but complex relationship between *HCN1* gene and WM.

Our results further supported the association between HCN1 gene polymorphism at rs1501357 and an impairment of working memory in schizophrenia patients, suggesting that HCN1 gene and HCN channel may be the genetic mechanisms of cognitive impairment in schizophrenia. It seems that, in addition to their antipsychotic effect, medicines targeting this gene may also improve cognitive symptoms of schizophrenia, especially WM maintenance, a common cognitive component across all three tasks used in Study 1. Consistently, Study 2 identified neural correlates of rs1501357 in the parietal cortex, a region that has been suggested to play a key and unique role in WM maintenance^10–12^. Specifically, previous studies have shown that brain activation in the parietal cortex increased linearly with the number of items until it reached its plateau when the number of items reached WM maintenance limit^13,14^. Two meta-analyses also reported altered parietal activation during the n-back task in schizophrenia patients^15,16^. Taken together, the risk allele seemed to be associated with parietal deficiency, consistent with a recent finding that parietal dysfunction was responsible for WM maintenance deficits of SCZ^17^.

The medial prefrontal cortex was the other region identified by Study 2. The medial prefrontal cortex was deactivated during the n-back task, consistent with its role as a key node of the default mode network (DMN)^18,19^. Numerous fMRI studies have suggested correlations between stronger deactivation in the DMN with increasing WM demands^20–23^. One study using single neuron recording also reported that the activity in the medial prefrontal cortex was related to WM maintenance in humans^24^. Patients with medial prefrontal cortex lesion were found to show impaired WM maintenance^25^. Patients with SCZ have also been found to show abnormal DMN activity^26,27^. Moreover, some of our own previous studies have suggested that the DMN may be a common region, through which risk genes could contribute to the etiology of SCZ^28–30^.

Although the gene-WM associations in patients with SCZ were consistent with our expectations and related literature, the opposite pattern for healthy controls was a surprise. There are no easy theoretical explanations for such results. This is mainly due to the fact that the functional consequence of the “C” risk allele to HCN channel expression/function is still unknown. However, animal studies on HCN channel have indicated the relationship between the same channel and working memory, which also showed a non-linear pattern. As suggested by Wang et al.’s animal study, HCN channel can affect the excitability of neurons by regulating the resting membrane potential. Low dose blockade can enhance neuronal firing, while high dose blockade can weaken neuronal firing^6,31^. It is then possible for rs1501357 to play opposite roles in patients with SCZ and healthy controls because HCN channel distribution varied across brain regions^32^ and its interaction with other cAMP-related proteins such as DISC1, PDE4A and D1R were different between patients with SCZ and healthy controls^31,33^. It is also worth mentioning that previous studies have reported similarly opposite patterns of results for patients with SCZ and healthy controls for other risk polymorphisms of SCZ^34–36^. For instance, rs10174400 within the *SCN2A* gene showed a clear allele dose-dependent effect on cognitive performance in SCZ but a weak opposite trend in controls^36^. Matsuzaka et al.^34^ reported that, compared to non-risk allele homozygotes, risk “G” carriers (rs165599 in *COMT* gene) obtained lower scores on a WM task in patients with SCZ but higher scores in healthy controls. These results are intriguing and worth further exploration. It highlights the importance of studying both patients with SCZ and healthy controls when exploring the functional impact of SCZ risk genes. It also suggests that medicines targeting the *HCN1* gene may produce differential effects on WM in patients with SCZ and healthy controls.

## Conclusions

In conclusion, this study used both behavioral and neural data to examine the association between a SCZ risk gene polymorphism (*HCN1* rs1501357) and WM maintenance. The association was opposite in patients with SCZ and healthy controls. These results expanded our understanding of the functional impact of the *HCN1* gene. Future studies need to systematically explore the contribution of this gene to other cognitive impairments of schizophrenia such as attention, executive function, and long-term memory.

## Materials and methods

The protocol of this study was reviewed and approved by the Institutional Review Board of the Institute of Cognitive Neuroscience and Learning at Beijing Normal University. All the participants were Han Chinese and gave their written informed consent for this study.

### Study 1: The behavioral study

#### Participants

The behavioral study initially included 852 healthy volunteers and 956 patients with SCZ. After excluding participants with missing data due to genotyping failures or other reasons, the final sample included 842 healthy volunteers and 876 patients. The majority of this sample came from our previous study^37^ which examined the associations between a polymorphism in the *NOS1* gene and performance on WM tasks (n-back and DPX tasks) and attention tasks (attention network test and Stroop task). In the current study, we focused on WM and hence only used the data from the n-back and the DPX tasks. Patients were recruited between August, 2008 and December, 2014 from the inpatients of the Ankang Hospital that was affiliated with Jining Medical University. All patients fulfilled the ICD-10 criteria for SCZ according to the diagnostic consensus of two experienced psychiatrists. Patients were excluded if one of the psychiatrists was uncertain about their diagnosis. The positive and negative syndrome scale (PANSS) was used to assess each patient’s positive (SAPS) and negative (SANS) symptoms. The mean score of the SAPS was 18.53 ± 6.94 and the mean score of the SANS was 17.53 ± 8.01. All patients were treated with a stable dose of atypical antipsychotics for more than 2 weeks, which included Clozapine, Olanzapine, Risperidone, Aripiprazole, Quetiapine, Ziprasidone, Haloperidol, Perphenazine, Sulpiride. Daily doses of antipsychotic medications were converted to standard chlorpromazine equivalents. Exclusion criteria for the patients included a history of other psychiatric disorders and severe brain injury (any closed or open injuries that may be related to current symptoms or cognitive functions), current substance abuse, currently having acute psychotic episodes, and failure to cooperate during the cognitive tests. The healthy controls were recruited from the residents living near Jining Medical University. All of them were interviewed by experienced psychiatrists to screen for any personal or family history of psychiatric disorders. Demographic variables are presented in Table 1.

#### Genotyping

Genomic DNA was extracted using the standard method. We used the Kompetitive Allele Specific PCR (KASP) (Shanghai Baygene Biotechnology Company Limited, Shanghai, China) method to genotype rs1501357. Ten percent of the samples were used for repeated detection, and the accuracy rate of the retest was 100%.

#### Cognitive tasks

Participants in this study finished 3 tasks: the 2-back task, the dot pattern expectancy (DPX) task, and the digit span task. All the tasks have been used in our previous studies^37–39^ and were administered using an IBM 14-inch screen notebook. Brief descriptions of all the tasks are presented below (see Supplementary Fig. S1 for more details).

The 2-back task included 48 trials. For each trial, a white circle was presented randomly at one of the four corners of a gray diamond-shaped square. Participants were required to recall the location of the white circle presented 2 trials before the current one. We used its accuracy to index the performance.

The DPX task included 160 trials. The stimuli were Braille font dot patterns and were presented in pairs (cue-probe). There were four conditions: AX (70% of total trials), AY (12.5%), BX (12.5%), and BY (5%), where A and B designate different cues and X and Y different probes. After 350 ms of a fixation cross, a cue was presented for 1000 ms, followed by a 4000-ms delay, and then the probe was presented for 500 ms. Participants were instructed to press a target key in the AX condition but to press the non-target key in all other conditions. Accuracy of the BX condition was used to index the performance.

The digit span task is a subtest of the Wechsler Adult Intelligence Scale-Revised (WAIS-R) which measures verbal WM. Participants were required to recall a list of digits in either forward or backward order and the length of the longest list recalled correctly was used to index verbal WM capacity.

#### Statistical analysis

Analyses were done using Statistical Product and Service Solutions software (SPSS version 22.0, SPSS Inc., Chicago, Illinois, USA). Non-genetic factors, including age, gender, and years of education, were compared across genotypic groups or between patients and controls using either ANOVA or the chi-square test. We used two-factor ANOVAs to examine the association between rs1501357 and WM performance. In these analyses, score for each WM task was entered as the dependent variable, whereas genotype and diagnosis were entered as independent variables. Post-hoc analyses were conducted when either the main effect of genotype or the genotype-by-diagnosis interaction effect was significant. The significance level was set at 0.05.

### Study 2: The fMRI study

#### Participants

The majority of the fMRI sample has been described in our previous studies^37,28,40–42^, which examined the effects of the *CACNA1C* gene (rs2007044), *MIR137* gene (rs1625579), *CAMKK2* gene (rs1063843), *NOS1* gene (rs3782206), and *ZNF804A* gene (rs1344706). The total sample consisted of 180 healthy volunteers and 65 patients, but only 155 healthy controls and 56 patients were included in the final analysis in this study after excluding participants with excessive head motion or failed genotyping. The patients were recruited between August 2008 and December 2017 from the inpatients of the Beijing Anding Hospital that was affiliated with Capital Medical University. All patients fulfilled the DSM-IV criteria (American Psychiatric Association, 2000) for SCZ according to the diagnostic consensus of two experienced psychiatrists based on a structured interview (SCID)^43^. The mean scores of the SANS and SAPS were 19.83 ± 6.96 and 20.14 ± 7.00, respectively. All patients were treated with a stable dose of atypical antipsychotics for more than 2 weeks. Daily doses of antipsychotic medications were converted to standard chlorpromazine equivalents. The types of drugs used were the same as in Study 1. Exclusion criteria were the same as for Study 1. Healthy controls were recruited by advertisement from Beijing and were interviewed by experienced psychiatrists to screen for any personal or family history of psychiatric disorders.

#### Genotyping

Genotyping was performed by the same KASP method as in Study 1.

#### fMRI task

As described in one of our previous studies^37^, participants were scanned with fMRI during a 2-back task. The stimulus was a white circle presented randomly at one of the four corners of a gray diamond-shaped square. Participants made responses according to the current location of the white circle (0-back) or the location of the white circle seen 2 trials earlier (2-back) using a fiber-optic response box with four buttons that were also arranged in a diamond shape. Participants pressed one of the four buttons to match the target stimulus. The task included two runs. Each run (lasting for 192 s) consisted of 8 blocks, in which the 2-back condition alternated with the 0-back condition. Each block started with a 4 s on-screen instruction (either the number “0” or “2” on the center of screen indicating the type of WM task to be performed). There were 8 trials in each block. In each trial (lasting for 2 s), the stimuli were presented for 500 ms, followed by a 1.5 s blank.

#### fMRI data acquisition

All imaging data were acquired at the Brain Imaging Center of Beijing Normal University. Participants were scanned using a Siemens Trio 3 T scanner (Siemens, Erlangen, Germany) with their head snugly fixed with straps and foam pads to restrict head movement. Functional images during task performance as mentioned above were collected axially using the following echo-planar imaging (EPI) sequence: repetition time (TR) = 2000 ms; echo time (TE) = 30 ms; flip angle (FA) = 90°; field of view (FOV) = 200 × 200 mm^2^; matrix size = 64 × 64; axial slices = 31; 4.0 mm slice thickness without gap (i.e. interleaved scan); voxel size = 3.1 × 3.1 × 4.0 mm^3^.

#### fMRI data preprocessing and analysis

Data preprocessing for fMRI data was implemented using Statistical Parametric Mapping software (SPM12, Wellcome Department of Cognitive Neurology, London, UK). Preprocessing included realignment (correcting for head movement; any subject with more than 2 mm translation or 2 degree of rotation was excluded), normalization (to the Montreal Neurological Institute (MNI) space), resampling (to a voxel size of 3 × 3 × 3 mm^3^) and spatial smoothing (with 8 mm full-width at half maximum (FWHM) of the Gaussian smoothing kernel).

After preprocessing, task condition (2-back vs. 0-back) was used as a predictor to produce brain activation images for each subject. A high-pass filter at 128 s was employed to remove noise associated with low-frequency confounds. The resulting images were entered into the second-level (between-participants) data analysis.

#### Statistical analysis

Owing to the small number of the TT genotype in patients, we combined the TT genotype with the TC genotype. One-way ANOVA and chi-square test in SPSS version 22.0 were used to compare genotypic differences on all demographic variables and behavioral data. We used 2 × 2 full factorial ANOVA in SPM 12.0 to test the associations. In this analysis, genotype (TT/TC vs. CC) and diagnosis (patients vs. controls) were entered as independent factors, and brain activation was the dependent factor. Significance was determined using an uncorrected voxel-level threshold of *P* < 0.001 and cluster-level family-wise error (FWE) corrected *P* < 0.05. Any significant genotype effect or genotype-by-diagnosis interaction effect on brain activation was followed up by post-hoc analysis using ANOVA.

## Supplementary information

Supplementary Figure S1

Supplementary materials
