# Supplementary figures and images for: Evidence for the contribution of HCN1 gene polymorphism (rs1501357) to working memory at both behavioral and neural levels in schizophrenia patients and healthy controls
Source: Schizophrenia (Heidelb). 2022 Aug 20;8(1):66. doi: 10.1038/s41537-022-00271-7 (PMC9392748; doi:10.1038/s41537-022-00271-7)

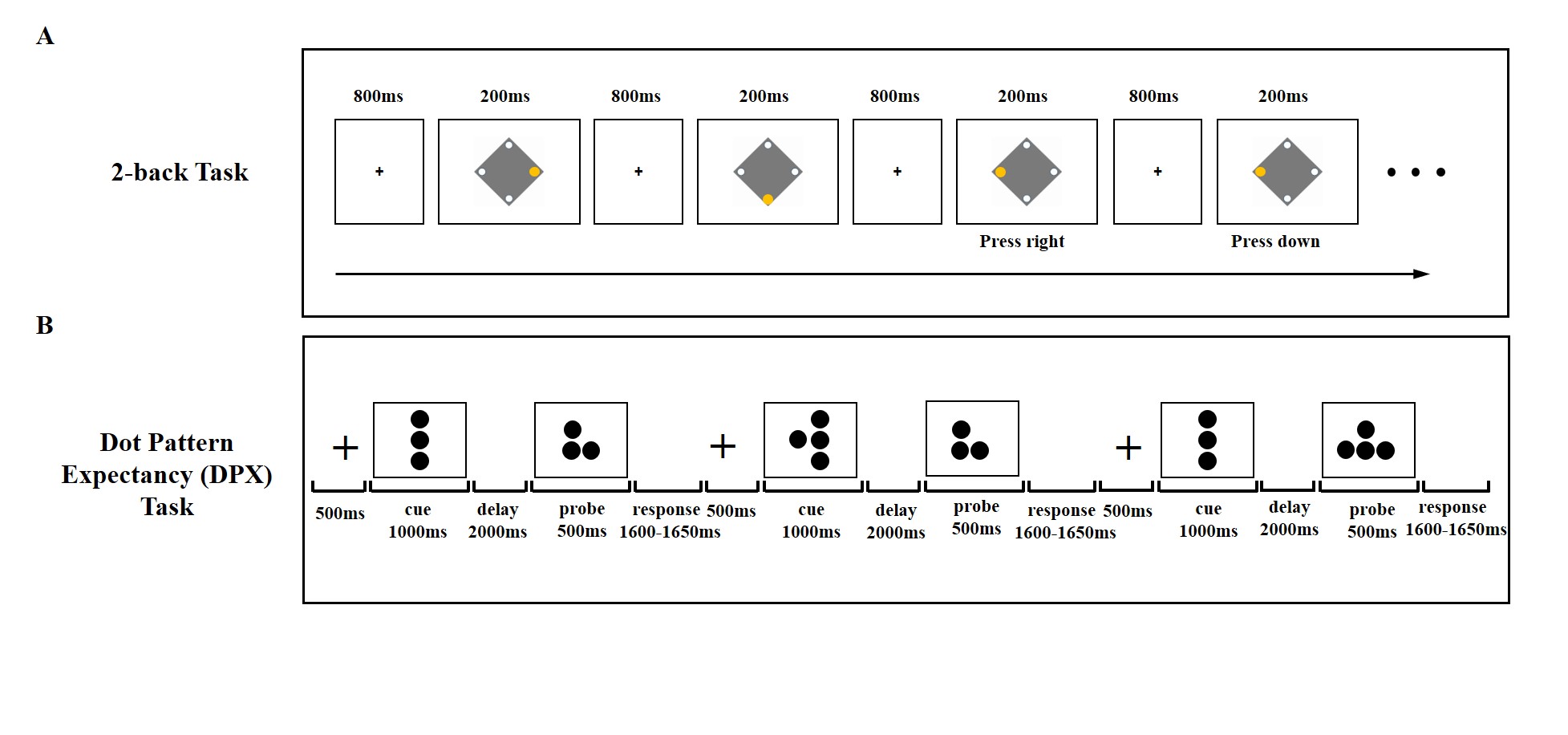

Supplement: Supplementary file 1 — Supplementary Figure S1 [file 41537_2022_271_MOESM1_ESM.jpg]
