# Supplementary material for: Evidence for the contribution of HCN1 gene polymorphism (rs1501357) to working memory at both behavioral and neural levels in schizophrenia patients and healthy controls
Source: Schizophrenia (Heidelb). 2022 Aug 20;8(1):66. doi: 10.1038/s41537-022-00271-7 (PMC9392748; doi:10.1038/s41537-022-00271-7)
Supplement: Supplementary file 2 — Supplementary materials [file 41537_2022_271_MOESM2_ESM.docx]

**Supplementary Materials**

**Data analysis**

In Study 1, three additional PGC2-reported SNPs (rs6466055, rs11210892 and rs4702) were also genotyped in this sample. We redid the ANOVAs to examine the association between rs1501357 and WM performance using these SNPs as covariates, which did not change our current results much (see Table S1).

Table S1. Working memory performance across rs1501357 genotypes

|  |  | Mean ± SD |  | F_diggnosis_ | F_genotypes_ | F_interaction_ |
| --- | --- | --- | --- | --- | --- | --- |
|  | TT | TC | CC |  |  |  |
| **DPX** |  |  |  |  |  |  |
| Controls | 0.80±0.29 | 0.82±0.28 | 0.88±0.22 | 182.648 | 0.260 | 5.533 |
| Patients | 0.62±0.26 | 0.63±0.31 | 0.57±0.31 | （0.000*） | (0.771) | （0.004*） |
| **Digit span** |  |  |  |  |  |  |
| Controls | 12.81±2.54 | 12.48±2.66 | 13.03±2.53 | 121.243 | 0.106 | 6.989 |
| Patients | 11.39±2.10 | 11.58±2.33 | 11.04±2.17 | (0.000*) | (0.899) | (0.001*) |
| **2-back** |  |  |  |  |  |  |
| Controls | 0.47±0.24 | 0.48±0.26 | 0.50±0.25 | 297.718 | 0.162 | 3.044 |
| Patients | 0.29±0.17 | 0.30±0.18 | 0.26±0.14 | (0.000*) | (0.850) | (0.048*) |

**ANT and Stroop task data analysis**

In Study 1, we additionally analyzed data about the attentional tasks (ANT and Stroop), which did not reveal any significant genotype effect or genotype-by-diagnoses interaction effect. These results were shown in Table S2.

Table S2. ANT and Stroop task performance across rs1501357 genotypes

|  | Mean ± SD | | | F_diggnosis_ | F_genotypes_ | F_interaction_ |
| --- | --- | --- | --- | --- | --- | --- |
|  | TT | TC | CC |  |  |  |
| ANT |  |  |  |  |  |  |
| Controls | 93.77±43.00 | 87.66±49.60 | 89.35±43.75 | 0.016  （0.899） | 0.728  （0.483） | 1.538  （0.215） |
| Patients | 90.97±63.93 | 95.34±76.48 | 85.73±88.64 |  |  |  |
| Stroop |  |  |  |  |  |  |
| Controls | 106.12±78.88 | 115.59±84.59 | 107.99±87.33 | 55.257  (0.000*) | 0.707  (0.493) | 1.224  (0.294) |
| Patients | 161.69±120.14 | 150.88±119.12 | 144.36±115.60 |  |  |  |

**Psychophysiological interaction (PPI) analysis**

In Study 2, we conducted psychophysiological interaction (PPI) analysis on task-dependent functional connectivity between the parietal cortex and the remaining brain, especially focusing on the prefrontal cortex. This analysis was also conducted using SPM12.

First, the seed VOI was defined as the coordinates where the genotype-by-diagnosis interaction effect was the strongest, within a 6-mm radius sphere centered on the interaction maximum (seed MNI coordinates: 30 -57 39). Then, the time series were extracted from the seed for each participant. Next, three regressors were used in the following GLM analysis, including the time series of the seed, the condition series of the task, and the interaction term between the first two regressors. Finally, the generated functional connectivity maps were entered into the group level analysis. we used 2 × 2 full factorial ANOVA, with genotype (CC vs. CT/TT) and diagnosis (patients vs. controls) as independent factors, and functional connectivity as the dependent factor. Significance was determined using an uncorrected voxel-level threshold of *P* < 0.001 and cluster-level family-wise error (FWE) corrected *P*_FWE_ < 0.05.

**PPI results**

We did not find significant genotype or genotype-by-diagnosis interaction effect between the parietal cortex and the prefrontal cortex. This indicated that our current finding about parietal cortex was independent to its functional connectivity with prefrontal cortex. By the way, we found significant genotype-by-diagnosis interaction effect within striatum (cluster size = 67 voxels, peak voxel MNI coordinates: -27 3 6, F = 16.29, *P*_FWE_ = 0.001).


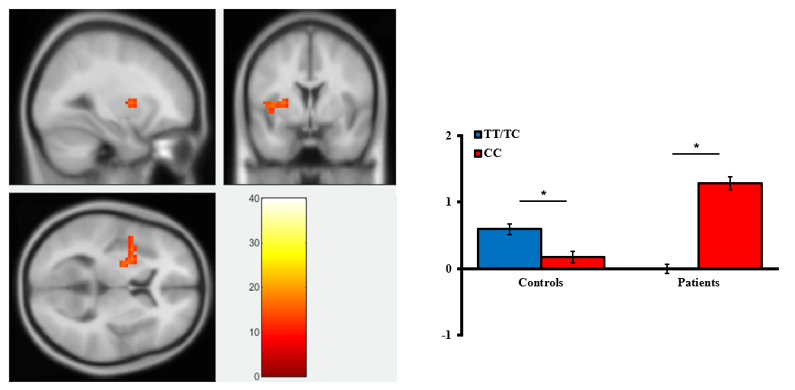


*

Figure S1. Genotype-by-diagnosis interaction on the functional connectivity between the parietal cortex and the striatum at 2-back task.
